# Hospital and Institutional News

**Published:** 1912-06-22

**Authors:** 


### HOSPITAL AND INSTITUTIONAL NEWS.

### THE "BIRTHDAY HONOURS."

FOREMOST among the medical and institutional recipients of "Birthday Honours" is Mr. Rickman Godlee, P.R.C.S., who is created a baronet. Mr. Godlee is a nephew of Lord Lister, and is Hon. Surgeon-in-Ordinary to the King, as well as Surgeon and Professor of Clinical Surgery at University College Hospital. Knighthoods are conferred upon Mr. F. Green, a deputy chairman of the League of Mercy; Mr. B. G. A. Moynihan, the well-known Leeds surgeon and pioneer of duodenal and stomach surgery; Mr. J. Bland-Sutton, surgeon to the Middlesex Hospital and to the Chelsea Hospital for Women; and Dr. St. Clair Thomson, throat surgeon to King's College Hospital. All these have well earned their respective honours by years of professional efficiency. Surgeon-Major-General A. P. Bradshaw, as we note elsewhere, becomes a K.C.B.; and the C.B. is conferred upon Colonel R. M. Campbell, of the I.M.S., and upon Surgeon-General W. Babtie, V.C. Then Professor Alexander Ogston, who is Surgeon-in-Ordinary to the King in Scotland and consulting surgeon to Aberdeen Royal Infirmary, receives the K.C.V.O.; and the C.V.O. is conferred upon Mr. G. L. Cheatle, surgeon to King's College Hospital; Dr. P. Horton Smith Hartley, assistant physician to St. Bartholomew's Hospital; and Inspector-General Belgrave Ninnis, R.N. The Fourth Class of the same Order goes to Mr. W. N. Barron, of Ascot, surgeon to the household of Prince and Princess Christian. Surgeon-General H. W. Stevenson, of the Indian Medical Service, becomes C.S.I.; and Lieut.-Colonel C. H. James receives the C.I.E. The only medical recipient of the Kaisar-i-Hind Gold Medal is Dr. M. R. T. M. N. Avergal, a Madras practitioner. One of the most interesting decorations in the list is the C.M.G. awarded to Dr. Andrew Balfour, of Khartum, a distinction as well merited as any that his Majesty has bestowed.

### THE CHEQUERED CAREER OF BANGOR INFIRMARY.

THE Infirmary Committee of the Bangor and Beaumaris Guardians have induced the latter to modify the proposed new workhouse infirmary. Originally the new institution was to cost some £17,000, and to provide for 124 beds. Mr. Bellis, the architect, has promised to modify the original scheme, and the effect of his instructions will be to dispense with two wings of the projected building and to reduce the number to 90. The amended plans will be ready by the 28th of this month, and the cost will be reduced to £13,000. It must be noted that the scheme for the new infirmary for one reason or another has suffered rather a chequered career. For instance, the tender for the original design had been already accepted by a builder of Todmorden, when this was found by mistake to have involved an addition of £1,000. It is to be hoped, however, that the scheme may now go forward, and that the Bangor Guardians may prove more lucky in their efforts than they can be said to have been hitherto.

### THE 1912 "BURDETT."

THE edition of "Burdett's Hospitals and Charities" for the current year is now published, and can be obtained from The Scientific Press, Limited, 28 Southampton Street, Strand, for 10s. 6d. net. Twenty-three successive annual issues have attested the value of this great work of reference, which contains information of more than 6,000 institutions—a fact of interest to those secretaries who have sometimes suggested that the figures in each volume should relate to the twelve months ending on December 31 immediately preceding its date of publication. If this had been possible it would have been done long ago. It is not possible for the simple reason that the 6,000 institutions concerned close their financial years on very varying dates. Where the same date is chosen and the number of institutions is small it is possible to give previous year's figures, as the King's Fund does in respect of the metropolitan hospitals, the accounts of all of which are made up to the same date in each year. In the new "Burdett" a special chapter is devoted to "The National Insurance Act and the Voluntary Hospitals," in which the danger attendant to hospitals on receiving grants in aid, and the way in which the voluntary system may be preserved and strengthened under the Act are explained in detail. Hospital architects will welcome the summary of hospital construction in 1911, which forms a chapter to itself. In addition, there are, of course, the usual features which have made "Burdett's Hospitals and Charities" the authoritative reference book of the voluntary hospitals and general charities of the Empire.

### A HOSPITAL SECRETARY KNIGHTED.

THE "Birthday Honours" this year have a special interest to hospital workers from the fact that among the promotions in and appointments to the Most Honourable Order of the Bath occurs the name of a hospital secretary. For many years the Oxford Eye Hospital has owed more than can be expressed in words to the keenness and affection which Surgeon-General A. F. Bradshaw, C.B., has displayed in its interests in his capacity of honorary secretary. Since his retirement from the Indian Medical Service General Bradshaw has found in hospital work an opportunity for his strenuous leisure, which has been the admiration of all who know him and who remember the long and distinguished career which he had previously in India. Having studied at St. Bartholomew's Hospital and qualified in 1856, General Bradshaw entered the Army Medical Department in 1857. He was present at the siege of Lucknow in 1858, and was garrison and brigade surgeon at Delhi during 1863. He held the post of principal medical officer of the Hazara (Black

Mountain) Field Force in 1891, when he was made a C.B., and of his Majesty's Forces in India from 1892-95. Among the very many congratulations which Sir Alexander Bradshaw will receive upon his promotion from Companion to Knight Commander none will be more sincere than those of the board, patients, and staff of the Oxford Eye Hospital. All of these have experienced those qualities of enthusiasm and kindness which have brought distinction to Sir Alexander in the three professions which he has adorned, those of soldier, doctor, and hospital secretary.

### A DOCTOR'S CRITICISM OF NEWSPAPER ADVERTISEMENTS.

For the first time, on Thursday, last week, Dr. Alfred Cox appeared before the Parliamentary Committee on Patent Medicines, and was still under cross-examination when the Committee adjourned. He stated that the British Medical Association realised that its campaign against patent medicines was peculiarly difficult, inasmuch as the press would not assist. The newspapers were so financially involved in the traffic by the charges they made for advertisements that it was no use, he said, looking to them to expose the real nature of the traffic. After affirming that only one weekly excluded quack advertisements altogether, Dr. Cox submitted that the provisions of the Sale of Food and Drugs Acts should be applied to proprietary medicines; that the Home Secretary should be empowered to institute proceedings against offenders; and that the Indecent Advertisements Act should be made to apply to advertisements of "female remedies."

### HOSPITALS AND SECRET REMEDIES.

At the further examination of Dr. Tirard, medical editor of the British Pharmacopœia, by the Committee on Patent Medicines, he gave his personal opinion that the Pharmacopœia should be produced at more frequent intervals, but if it were revised every time criticisms were offered untold confusion would result. The proper laboratory for ascertaining the medicinal value of drugs was, he said, the hospital, where an accurate knowledge of the value of drugs was obtained as a result of continual observation. The hospital was in fact the only laboratory for testing, but the out-patient department he considered absolutely useless for the purpose of accurate information.

### STAFF ELECTIONS AT CHARING CROSS HOSPITAL.

The appointment of assistant obstetric physician at Charing Cross Hospital has been filled by the election of Mr. Cuthbert Lockyer, M.D., F.R.C.S., M.R.C.P. This appointment is especially interesting because at the time of the last vacancy, fourteen years ago, Dr. Lockyer was recommended by the medical committee, but was passed over by the governing board in favour of Dr. T. W. Eden. In the interval Dr. Lockyer has continued to serve his hospital as obstetric tutor; and has also become a member of the honorary staff at the Great Northern, Samaritan, and British Lying-In Hospitals. The vacancy at Charing Cross Hospital for an assistant surgeon has been filled by the appointment of Mr. W. Fenwick.

### TYPHOID PROPHYLAXIS.

After many disappointments, the prevention of typhoid fever on Wright's principles seems to have been brought so near perfection that there can no longer be any doubt as to its value. In the United States anti-typhoid vaccination has now been made compulsory. When large numbers of American troops were assembled for the invasion of Cuba, typhoid fever broke out and carried off hundreds of victims. In a similar latitude and climate, United States troops were mobilised on the Mexican border three years ago, and 12,800 of them were vaccinated: two cases of typhoid occurred in this large division in four months, neither of them fatal. Since then more statistics have become available, and it suffices to quote them from an American army surgeon in the *American Journal of the Medical Sciences*. Among 17,978 vaccinated soldiers, seven had typhoid fever, with no deaths. Among 58,252 non-vaccinated soldiers there have been in the same time and in the same circumstances 135 cases with ten deaths.

### OPENING OF THE NELSON HOSPITAL.

Preceding the opening of the Nelson Hospital for Wimbledon and Merton on Friday last week, a pound day, organised by ladies interested in the hospital, was held. Donors were allowed to inspect the new hospital and a continuous procession came from morning till late evening. The result was that the store-room shelves were heaped with large quantities of tea, sugar, and rice, besides many gifts of crockery, numbers of chairs, and some blankets. A florist sent a stock of geraniums and other plants for the garden, and another supply of plants was received from Wimbledon Parish Church Flower Service. £10 in money was presented. The gifts were received by the Matron, Mrs. Deas, Mrs. Randall, Miss Carver, and other ladies. In addition, forty men who have been engaged on the building were entertained to a supper at the Masonic Hall, Merton. The Chairman of the Hospital Committee, Mr. T. C. Summerhays, J.P., presided. The hospital, as we have previously noted, is a memorial to Lord Nelson and is erected in the parish in which he lived previous to sailing on his last cruise. A medallion of the Admiral carved in oak from H.M.S. *Victory* has been placed in the board room and his bust in the main corridor. The famous signal "England expects" was, on the opening day, flown outside the hospital, and it is believed that the flags which had been lent for this purpose are the only set in England.

### THE NEW ISOLATION WARD AT PENDLEBURY.

A new isolation block containing eight beds for infectious cases and for those patients needing isolation and observation was opened on Wednesday last week at the Manchester Children's Hospital by the president, Sir George Wm. Agnew, Bart., M.P., in the presence of a large number of Governors and members of the Ladies' Auxiliary Fund. A vote of thanks to the president for performing the opening ceremony was proposed by Mr. Wm. Buckley, J.P., chairman of the House Committee, and seconded by Mr. George Comber, J.P., hon. treasurer. The visitors afterwards inspected the new building, which is of wood lined inside with felt, and is a self-contained unit, containing six beds for patients and two beds for nurses, with bedroom accommodation for two isolation staff nurses and a servant. Two separate projecting blocks, giving cross-ventilation, contain lavatory and bath accommodation for both patients and staff. The roofs are covered with the new "Ruberoid" patent covering of grey colour. The cost will be about £1,600, which includes the brick pier foundations, and the laying-out of adjacent ground and the making of roads, etc. The Chairman of the Board, Mr. Arthur Heywood, made an earnest appeal for funds towards the cost of this new and necessary extension. Additional accommodation for the nursing staff is also being provided by extending the present nurses' home on the south side of the hospital. The contract has been let and the work is now rapidly proceeding.

### PUBLIC INTEREST IN MANCHESTER CHILDREN'S HOSPITAL.

It is interesting to add that about sixty delegates and members of the Executive Committee of the Manchester and Salford Hospital Saturday Fund visited the Manchester Children's Hospital at Pendlebury on Saturday last. After the party had been photographed in the grounds they were shown over the hospital by Mr. John Dendy and Mr. G. A. T. Jesson, members of the Board of Governors. Replying to the welcome extended to the visitors by Mr. Dendy on behalf of the Board of the hospital, Mr. C. Swinglehurst, chairman of the Executive Committee of the Saturday Fund, expressed the pleasure which the visit had given to those present, and stated that he felt a visit such as this would greatly increase the interest taken by the workpeople in the voluntary hospitals. About two hundred members attending the Tuberculosis Conference sitting in Manchester also visited the hospital, and went round the wards and various departments.

### "SYNTHETIC RUBBER."

The announcement made by Professor W. H. Perkin, F.R.S., at a meeting of the London Section of the Society of Chemical Industry on Monday of this week, to the effect that a process had been discovered by which rubber, equal to natural rubber, could be produced synthetically is of peculiar interest to hospital authorities. The cost of the many appliances composed in part, or wholly, of rubber is an important item, more especially when the prices advance to the figures reached during the recent rubber boom. At that time the best rubber fetched as much as 10s. per lb., and medical and surgical appliances composed of rubber advanced in price to a proportionate extent. It is estimated that synthetic rubber can be produced to sell at a profit at 2s. 6d. per lb., and possibly at a much lower figure. The present price of the finest Para rubber quoted in Mincing Lane is from 4s. 9d. to 5s. per lb., while the possibility of a recurrence of the late extravagant prices is rendered practically impossible.

### HOSPITALS AND THE TELEPHONE TRANSFER.

In the House of Commons on Monday Mr. Stuart-Wortley, M.P., asked the Postmaster-General: Whether the National Telephone Company used to grant to hospitals reduced rates, and whether, since the transfer to the State, these favourable terms had been in all or any cases withdrawn; and, if so, whether it was intended to persist in such withdrawal. Captain Norton, in reply to this, said that the National Telephone Company came to the conclusion that such reductions ought not to be allowed, and they began to discontinue the practice some years ago. A general obligation, he continued, is imposed upon the Postmaster-General by statute not to show favour or preference; and this obligation requires him to provide telephone services on uniform terms. In short, it appears that hospitals have not gained by the transfer, except possibly in the virtue of patience, which has recently been severely tried by a somewhat disorganised service.

### A "MARMALADE" SERVICE.

A service for children of a somewhat novel character is an annual institution at St. Gabriel's Church, Walthamstow, and is popularly known as a "Marmalade" Service. It was started some four years ago by the then curate-in-charge, the Rev. S. C. Rees-Jones, who, realising the practical help that might accrue to the local General Hospital, proposed that, instead of a Flower Service, a "Marmalade" Service should be held. Every child in the Sunday School is asked to bring a jar of marmalade to the special service, with the result that this service has added considerably over 100 lb. weight of marmalade every year to the stores of the Walthamstow Hospital. The promoters suggest that such a service might be adapted to local conditions and needs in many parishes, and thus be the means of considerably helping the local hospitals.

### THE DOCK STRIKE AND OUT-PATIENT DEPARTMENTS.

While the strike does not appear to have affected the economic departments of the hospitals to any serious extent, the out-patient departments of those institutions which are in the neighbourhood of the centres of its chief activity have been called upon to treat more than the average number of adult male patients. Probably the reason is that the enforced holiday consequent on the issue of the order to "down tools" gave the men the opportunity to seek medical advice for trivial complaints. Ordinarily a dock labourer cannot afford the time to go to an out-patient department for a minor defect that neither worries nor unduly troubles him. At such a time as this, however, he has leisure to take an interest in the triviality—at least until he is told that treatment may mean operation and further rest in bed. A house surgeon at one hospital states that the percentage of varicocele, varicose veins, and lipomata in adult males visiting his department has suddenly jumped up 20 per cent. during the last two weeks. The cause of this increase is not a multiplied incidence of these lesions, but simply the leisure afforded by the strike itself. Those who are responsible for the stoppage of food supplies and general transport would do well to bear in mind that they run a risk of harming themselves in one point of hospital treatment at least. During strikes, when the elementary precautions necessary to prevent a breakdown in the institution do not permit the admission of trivial cases into hospital nor, scarcely, the performance of "operations of convenience," these numerous out-patients must be sent back and told to come up again in a month, or when circumstances are not so pressing. The strike is probably responsible for the fact that such patients realise the desirability of having their lesions attended to, but it also produces the result that they cannot usually be treated as in-patients and relieved of their troubles.

### THE "MILWARD" BED AT THE BIRMINGHAM GENERAL HOSPITAL.

The dedication plate of the bed in memory of the late Mr. Frederic Victor Milward was unveiled in Ward 5 at the General Hospital, Birmingham, last week. This bed has a special and sad significance from the fact that Mr. Milward was assistant surgeon to the hospital when he died just over two years ago. The unveiling was performed by Mrs. Victor Milward and Mrs. F. V. Milward, and, in addition to the other relatives present, the House Committee was represented by the Deputy Chairman, Mr. John Henry Lloyd, who thanked the family for their generous gift, Sir Robert Simon, Mr. F. S. Goodwin, Mr. B. B. Tilley, and others.

### A NEW SANATORIUM AT IPSWICH.

On Friday last week Lord Balfour of Burleigh declared King Edward the Seventh's Ipswich Memorial Sanatorium open. He congratulated the Mayor and Corporation on the progress they had already made by the institution of tuberculosis dispensaries. Alderman W. F. Paul, the Chairman of the Sanatorium Committee, stated that the whole sum required—over £15,000—had been voluntarily subscribed, and on behalf of his committee handed over the building debt free to the town of Ipswich. The Mayor (Alderman Rands), in formally accepting the Sanatorium, recalled that Dr. A. M. N. Pringle, Medical Officer of Health of the Borough, first mooted the subject in his report two years ago, and that Alderman Paul, the chairman of the Public Health Committee, had subscribed £5,000 to the initial expenses. The architect is Mr. H. Munro Cautley. A vote of thanks was then given to Mr. E. G. Pretyman, M.P., the donor of the site. The Sanatorium stands on a site of 16½ acres of beautiful undulating heathland—part of the Orwell Park Estate—two miles from the town and on higher ground. The land in front is open heath, whilst at the rear it is sheltered by a pine wood. The subsoil is of sand, and the water supply is from the town mains which have been extended at the expense of the Corporation. The Sanatorium proper is a two-floor brick building constructed in the form of a crescent and gives accommodation for sixty-five patients. The administration block is entirely detached from this building and stands to the rear or north. The patients' bedrooms lead out on to balconies, the doors being fitted with louvres to admit a sufficient supply of fresh air in wet weather. The buildings throughout are lighted by electricity, and the generating steam plant ensures a supply of hot water and steam for other purposes. The committee did not put forward their scheme until they had made themselves acquainted with the sanatoria of the country by personal visits. Frimley, of course, was one of the places visited, and it is perhaps not to be wondered at that their first medical superintendent should be the late assistant medical superintendent at Frimley Sanatorium, Mr. W. F. Sutcliffe, M.R.C.S., L.R.C.P., who took up his duties at Ipswich on May 14.

### THE MAURICE MEMORIAL AT MARLBOROUGH.

A subscription list has now been opened for raising a memorial to the late Dr. J. B. Maurice, whose long public connection with the town (and College) of Marlborough and whose work in connection with the Savernake Cottage Hospital were described in our columns at the time of his death some weeks ago. The Mayor of Marlborough, Alderman T. Free, is taking the matter up, and it may confidently be expected that Marlborough and the district will combine to raise a fund worthy of its once most prominent and best-loved citizen.

### THE MILNE TREATMENT IN GERMANY.

It will be remembered that unpleasantness arose a few months ago between the lay controllers of a British hospital and the resident medical officer because the latter resented an attempt by the former to dictate to him over a matter of treatment—namely, the Milne system for scarlet fever. Lately this treatment has been tested by Koerber at the Hamburg-Eppendorf Hospital. This observer arranged to treat one-half of those admitted during a certain period in the ordinary way, and the other half with eucalyptus and carbolic acid according to Milne's routine. He found that the mortality of the two series was 2.56 per cent. and 2 per cent. respectively, this slight advantage resting with the Milne treatment. Endocarditis and nephritis were also less frequent in the Milne cases, but arthritic complications were more numerous than in the control patients. He also concluded that the spread of infection is not inhibited, as Milne claims it is, for "return cases" were slightly increased in number. Nor was the average length of the disease diminished.

### THE NEW CHAIRMAN OF THE WEST END HOSPITAL.

Mr. Leonard Brassey, M.P., J.P., has been elected chairman of the West End Hospital for Diseases of the Nervous System. Mr. Brassey and Lady Violet Brassey, who is president of the Ladies' Guild in connection with the hospital, have taken a great interest in the work and have for some years given a summer convalescent home for patients of the hospital.

### THE LEEDS INFIRMARY EXTENSION SCHEME.

It is announced that a committee representing all parties on the Leeds City Council has given its approval, subject to certain modifications, to the great extension scheme of the Leeds General Infirmary which we summarised last week. The proposed modifications are being made the subject of negotiation.

### A TEN O'CLOCK RULE AND AN ISOLATION HOSPITAL.

At a recent meeting of the Keighley and Bingley Joint Hospital Board, Mr. W. Coleman moved that no member of the board should attend the hospital after seven in the evening. He stated that information had reached him that several members stayed there until ten o'clock at night, and he thought it a shame that they should do so. The motion found no seconder and the subject dropped. It is a pity that it was allowed to do so until the veiled charge which the resolution implied was either specifically stated or disposed of. But just as hearsay statements are no evidence in a court of law, so are they to be deprecated at public meetings.

### A HOSPITAL CHAIRMAN DEFENDS HIS COLLEAGUE.

Mr. John M'Meekan, J.P., chairman of the Bangor Cottage Hospital, took occasion at the recent monthly meeting to defend the honorary treasurer, Mr. C. H. Bowen, from certain attacks which were made on him at the annual meeting. Mr. M'Meekan assured those present that the attack was quite unjustifiable so far as Mr. Bowen and the committee were concerned. "I have told Mr. Davidson," the Chairman proceeded, "that if he has a grievance he should have brought it up at the executive committee, and if he failed to get redress there, then he could have brought it before the annual meeting." The executive committee, he thought, could only express their thorough confidence in Mr. Bowen's integrity. At a subsequent meeting of the general committee Mr. M'Meekan was re-elected chairman and Mr. C. H. Bowen honorary secretary.

### AXMINSTER'S NEW COTTAGE HOSPITAL.

We publish elsewhere an architectural description of the new buildings into which the old institution at Axminster is moving, and which were opened on Tuesday last by Mrs. Conybeare Craven, who founded the hospital in 1886. The building subcommittee have placed on record the patience and enthusiasm with which Mr. Hoel Hacon, the honorary secretary, has worked during the transition. It was largely through his courtesy and information that The Hospital was able last autumn to publish an account of the development of this cottage hospital, the origin of which has earned a place in history from the fact that its first home was the original factory at which the township's famous carpets were made. We wish the institution every success in its new buildings, which mark the growing importance of the work which it is called upon to do.

### THE DUTIES OF A MEDICAL SUPERINTENDENT.

The statement that the existing rules and the by-laws nominally in force at the Dundee Royal Infirmary were in fact a dead-letter caused much discussion at the recent annual meeting, when several revised by-laws were adopted. In recommending them Mr. T. H. Smith said that the regulations imposed on the medical superintendent medical and surgical duties impossible for him to undertake and which had of necessity fallen into abeyance. The visiting staff, he continued, should have full responsibility for the care and treatment of patients, while the discipline and conduct of the institution should be, as hitherto, the prerogative of the superintendent. The revised by-laws, he added, provided for a medical committee. Mr. T. H. H. Walker and Mr. J. M. Hendry supported the revised by-laws, the latter remarking that none of the superintendent's duties would be taken away, and that the existing rules were ignored completely. Mr. P. S. Brown thought that the superintendent should exercise a general control over the resident officers in the treatment of patients. Mr. A. B. Gilroy urged delay, and said that the public would not approve of a change which would not permit the superintendent to attend urgency cases unless allowed by the house surgeons. With this view Mr. James Guthrie agreed, but after Mr. Walter Shepheard, the chairman, had advocated postponement, voting took place and the new by-laws were carried.

### LORD HALDANE AND THE GERMAN HOSPITAL.

The annual festival dinner of the German Hospital, Dalston, takes place next week. Usually the hospital dinner of the "German" is one of the most enjoyable of institutional dinners in London. There is invariably an attractive programme, the speeches are short and to the point, and it supplies what Talleyrand called the "indispensable characteristic of a good dinner," in that it is purely a male dinner, no ladies being present. There is always a good collection to report—a collection made by the stewards months beforehand, so that no one attending the function is harassed by the thought that he may be called upon, immediately after having eaten his entremet, to subscribe to the funds of the hospital—and generally an interesting record of progress to be detailed. This year the chairman of the dinner is to be the new Lord Chancellor. Lord Haldane, as is well known, takes an interest in German art and literature, and he is said to be a keenly appreciative critic of what has been done in the Fatherland in the matter of hospitals. Perhaps, though we are not certain on this point, he is the only member of the Cabinet who thoroughly knows what the hospitals in Germany and the German Hospital in London have done to merit the high praise that has been bestowed on them. His chairmanship this year at the festival will be doubly important, for the Dalston institution is just emerging from a reconstruction scheme which has made it much more up to date than it used to be. It has recently received several valuable donations and legacies, among them Sir Julius Wernher's bequest of £20,000. Sir Julius, it may be added, was always a good friend of the hospital and contributed liberally towards its funds. The Dalston Hospital is one of the few general hospitals in London that can boast of being clear of debt. This satisfactory state of affairs is largely owing to the fact that the annual festival dinner brings in sufficient funds to wipe off the deficit remaining when the donations and subscriptions have failed to equal expenditure.

### ST. PAUL'S EYE HOSPITAL, LIVERPOOL.

It is now very nearly a year since St. Paul's Eye Hospital moved into its new buildings in Oldhall Street. A move had long been contemplated, and its cost up to the present has amounted to about £15,500. About four-fifths of this has been raised by friends of the institution, nearly £4,000 having been specially devoted to the George Edward Walker Memorial Children's Department in memory of the hospital's founder. It should be remembered that the change of site raised the number of beds from forty-four to sixty-eight. Mr. F. Stubbs is urging that the deficit should be paid off, and at the annual meeting last week Lord Derby and Sir William Bowring were among the speakers.

### NEW IMPROVEMENTS AT WORCESTER INFIRMARY.

The question of a new $x$-ray apparatus was again brought up at the monthly meeting of the executive committee of Worcester General Infirmary, when the report of Dr. Hall Edwards, who had been asked to act as advisor, was received. He stated that the present installation was old, of an obsolete design, and quite inefficient. With the proposed installation, he thought that the infirmary would be as efficiently equipped as any London hospital. The estimate of £345 was accepted. Feeling strongly that some better system than the present was called for, further expenditure for the lighting by electric light of the whole infirmary was discussed and an estimate of £125 was accepted by the committee. It was also decided to provide telephonic communication between the wards and other rooms in the infirmary, at an estimated cost of some £26.

### THE RENOVATION OF CHESTER INFIRMARY.

The offer of Mr. Alfred Wood to contribute £12,500 so that two wings may be added to the institution has encouraged the Governors immediately to renovate their buildings, as the board of management explains in an appeal. The entire scheme is expected to cost £40,000, and the statement having been previously made that "the extent to which the renovation can be carried out is entirely a matter of finance," Mr. Wood's offer has peculiar importance. We understand that the new out-patient department has already found a sponsor, and we hope that the example so early set by the Pageant Committee, and since followed up, will prove again the old assertion that "nothing succeeds like success," as all must do who have an expert knowledge of the problem before the Chester Infirmary.

### A SURGEON'S TRIBUTE TO WOMEN.

Those women who decried a famous pathologist's recent indictment of their sex had their revenge at the annual festival dinner of the Kensington and Fulham General Hospital last week, when Dr. Cyril Horsford, aural surgeon to the hospital, urged the importance of forming a ladies' association. His appeal was remarkable for the emphasis which he laid on women's power to bring the work of the hospital to the notice of a larger public. His view in fact was that women were more useful in this direction than men. He did not suggest that the ladies' association should do more than advertise the hospital and beg for funds, but in view of the good work in other directions that has been done by many ladies' associations attached to the great hospitals we hope that one will be formed in connection with the Kensington and Fulham General Hospital. Lord Claud Hamilton presided at the dinner, and Dr. Macnaughton explained the need for reopening the children's ward, which has been closed for want of money.

### THIS WEEK'S DRUG MARKET.

The labour troubles at the docks still overshadow the Drug market, and while it would be an exaggeration to write that business is at a standstill it would certainly be true to describe it as very depressed. The public auction of drugs which should have been held in Mincing Lane last week has had to be postponed until June 27, and there is some doubt whether the difficulties of obtaining drugs from the wharves, etc., will have been overcome before that date. A few price changes have taken place. As was anticipated, makers of calomel, corrosive sublimate, and other salts of mercury have reduced their prices by one penny per pound as a result of the reduction in the value of quicksilver. Contrary to expectation prices of iodine and iodides have been materially advanced; a reduction rather than a rise was expected, so that the higher prices come as a surprise. Opium and morphine continue to show a slightly advancing tendency. The price of cocaine hydrochloride has been reduced by ninepence per oz.—a movement which was not unexpected. A further advance in the quotation for santonin is regarded as probable. Rhubarb is slightly dearer, as also is balsam tolu. Eucalyptus oil is tending upwards in price. Ergot of rye tends lower.

### TO OUR READERS.

Contributions are specially invited from any of our readers to these columns. They should deal with topical subjects and news. They must be authenticated for the information of the Editor only. The minimum payment if published is 5s. There is no hard-and-fast rule as to space, but notes of about twenty lines in length are preferred.

